# Advancing statistical learning and artificial intelligence in nanophotonics inverse design

**DOI:** 10.1515/nanoph-2021-0660

**Published:** 2021-12-22

**Authors:** Qizhou Wang, Maksim Makarenko, Arturo Burguete Lopez, Fedor Getman, Andrea Fratalocchi

**Affiliations:** PRIMALIGHT, Faculty of Electrical Engineering, King Abdullah University of Science and Technology (KAUST), Thuwal 23955-6900, Saudi Arabia

**Keywords:** deep learning, inverse design, metamaterials, nanophotonics, optimization

## Abstract

Nanophotonics inverse design is a rapidly expanding research field whose goal is to focus users on defining complex, high-level optical functionalities while leveraging machines to search for the required material and geometry configurations in sub-wavelength structures. The journey of inverse design begins with traditional optimization tools such as topology optimization and heuristics methods, including simulated annealing, swarm optimization, and genetic algorithms. Recently, the blossoming of deep learning in various areas of data-driven science and engineering has begun to permeate nanophotonics inverse design intensely. This review discusses state-of-the-art optimizations methods, deep learning, and more recent hybrid techniques, analyzing the advantages, challenges, and perspectives of inverse design both as a science and an engineering.

## 1 Introduction

Throughout the past decade, the complexity of nanophotonics circuitry increased exponentially, mixing non-linearity with dense nanoscale integration, advanced material manufacturing, and broad-band engineering functionalities [1–3]. Traditional intuition-driven design based on electromagnetic (EM) simulations manifests several weaknesses for modern device engineering that could efficiently incorporate all the factors mentioned above. While lacking clear demonstration that one preferred design is optimal against all required constraints [4], direct design often relies on brute-force searches, leading to either high computational costs or oversimplifications on design domains [5]. Inverse design, a paradigm in which the user provides the desired output, and the machine finds the required geometry, promises to address these bottlenecks by furnishing an automated platform for nanophotonic systems engineering [6].

**Figure 1:**
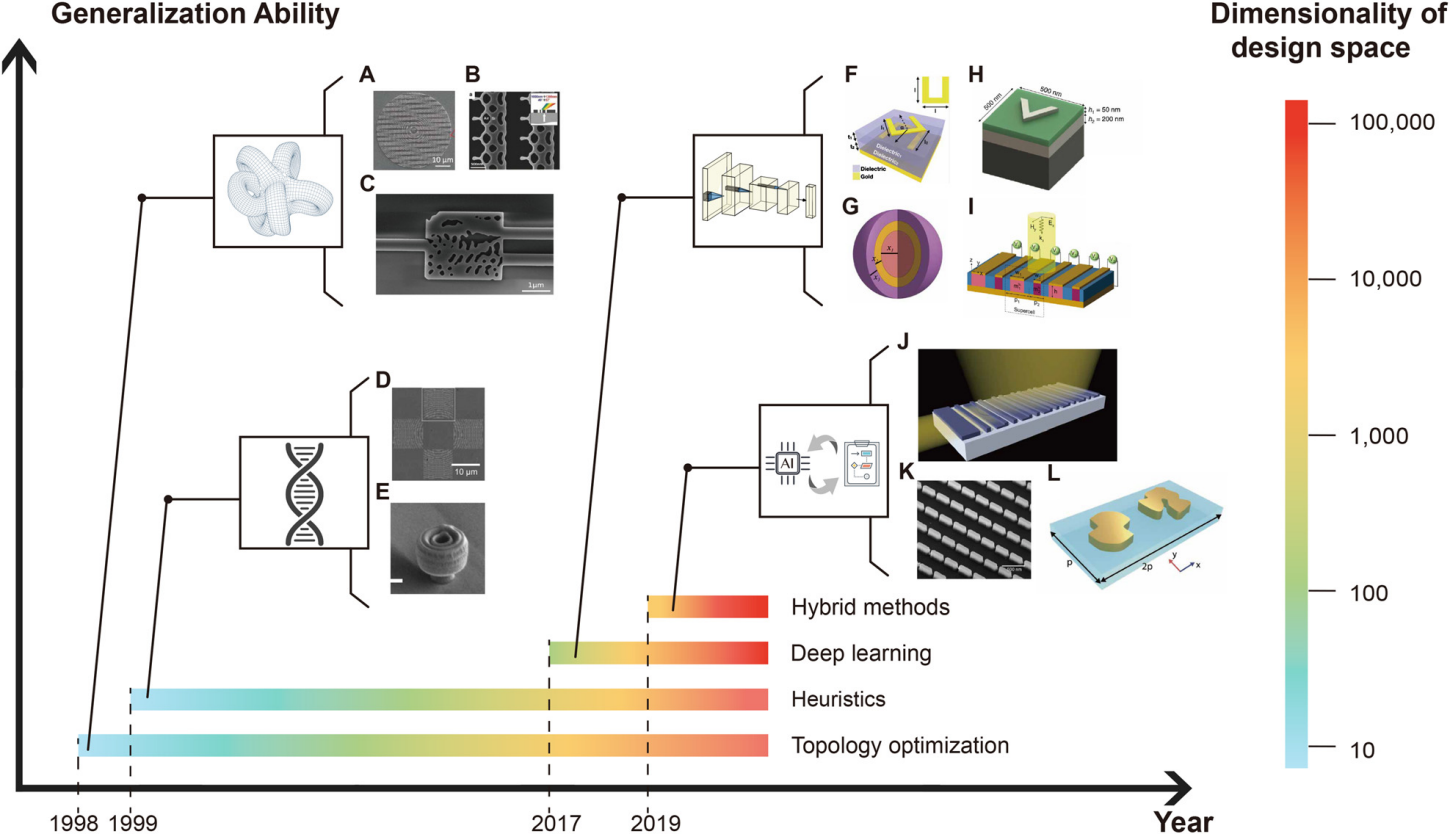
Schematic introduction of inverse design. The figure compares four main sub-fields of inverse design in terms of generalization ability and dimensionality of design space. From the bottom to top, the introduced approaches are respectively: **Topology optimization** which utilizes gradient-based numerical iteration tools on the device shape, often represented by the spatial distribution of permittivity/refractive index. The exhibited applications include: (A) an amorphous silicon metalens. Adapted from [34]. (B) A broadband blazed metagrating. Adapted from [29]. (C) A wavelength demultiplexer operation at 1300 nm and 1550 nm. Adapted from [27]. **Heuristics** which imitates natural phenomenon and solves non-gradient based optimization problems. The exhibited applications include: (D) a metasurface with on-demand focal length composed of lattice opto-materials. Adapted from [132]. (E) A high-NA nanophotonic lens for GaAs nanowires. Adapted from [133]. **Deep learning** which trains neural networks to model the dual relationship between design parameters and corresponding optical responses. The exhibited applications include: (F) on-demand design of chiral metamaterials. Adapted from [134]. (G) Prediction of scattering coefficients of eight-shell nanoparticles. Adapted from [135]. (H) Design of reflective silver nanostructurs. Adapted from [110]. (I) Hybrid metasurfaces composed of plasmonic structures and phase-change materials. Adapted from [118]. **Hybrid methods** which combines both advantage from deep learning and optimization-based methods, resulting in accelerated iteration and less requirement of the dataset. The exhibited applications include: (J) vertical grating couplers working in the C band. Adapted from [16]. (K) Flexible metasurfaces for arbitrary resonance control. Adapted from [19]. (L) Metallic metamolecules for polarization rotation. Adapted from [17].

Initial applications of inverse design (Figure 1) rely on classical numerical optimization algorithms [7, 8], employing suitably defined cost functions that the scheme minimizes after successive iterations. The minimum of the cost function defines the design’s objective and identifies a set of parameters that specify the design sought. The main drawback of this type of inverse design is the lack of generalization ability: all the information learned during the search of one design is typically lost and not used in the future, requiring the designer to rerun the optimization. The recent development in artificial intelligence and deep neural networks (NN) [9, 10] is significantly reshaping this field, implementing new schemes that take advantage of the universal learning and prediction abilities of NN.

NN are statistical learning systems composed of networks of sequentially layered neurons, with each neuron processing a weighted sum of forehead layers through a non-linear activation function [11]. In theory, sufficiently deep NN layers have universal approximation abilities: they can learn a user-defined function from the analysis of input–output data sequences and perform predictions on future trends [12]. Such generalization ability originated new inverse design schemes that can predict novel device configurations by forwarding queries on the NN, with a generalized framework that easily transfers between similar design tasks [13]. A challenge lies in the chaotic instabilities of NN models, which sometimes arise because of the model complexity [14].

Recently, different research groups have proposed hybrid strategies combining both deep learning and optimization methods to overcome this issue [15–19]. These methods leverage sophisticated deconstruction approaches when changing design parameters during the iterative trial-and-error search process [18]. Hybrid schemes, the most recent trend in inverse design, are generally capable of training large spaces of design parameters while preserving the productivity among transferred tasks, thus retaining a strong generalization ability within highly complex design spaces.

## 2 Inverse design by optimization

These inverse design approaches leverage classical optimization techniques to explore the design space of possible solutions efficiently, ideally converging to the desired result exponentially faster if compared to a direct search [4]. The main idea of these methods is to define a suitable figure of merit (FOM), or equivalently a cost function whose minimum defines the structure sought to engineer. The cost function is progressively optimized with an intelligent exploration of its landscape by either constructing approximate models or exploring neighboring manifolds in the design space. The main difference between these two approaches is merely the possibility to differentiate the cost function with various choice of design variables. When the parameters are distributed in continuous ranges, such as width, thickness, refractive index, and hole sizes, the gradient is computationally available, and topology optimization [20] provides a fast and robust search with a mathematically guaranteed rate of convergence in a local minimum of the FOM. If the problem considered is intrinsically not differentiable, for example, considering discrete material categories with characteristics selected from a library of candidates, heuristic optimization [21] will be a preferred choice. Although sometimes lacking a precise theory of convergence, these nature-inspired approaches can identify global minima of the cost function, even in large designs spaces where the objective function contains an exponential number of local, sub-optimal solutions.

### 2.1 Topology optimization

Gradient-based inverse designs exploit the first-order derivative (gradient) of the cost function to build approximate models that are minimized at each iteration [22–38]. Traditional topology optimization implements the gradient-descent optimization scheme [39, 40], while advanced techniques exploit convex optimization such as the trust-region [41], and moving asymptotes [42, 43]. In topology optimization based on density variables, the design parameters ***β*** are typically the permittivity *ϵ*_*r*_(**x**) probed at discrete spatial lattice points **x**. The resulting design space is a 2D binary image, in which the pixel density measures the distribution of different classes of materials [27–34]. Other topology approaches express ***β*** following a re-parameterization strategy such as, e.g., the level-set method [36–38]. In two-dimensional material profiles, the level-set method represents design variables via horizontal slices of a 3D surface, defined as the level-set function. During the optimization, the continuous movement of a cross-sectional plane simulates the variation of design variables. In contrast to explicit curve representation such as analytic equations, the level-set method traces topology changes, including merging, splitting, generating, and vanishing different shapes.

In topology optimization, the iterative calculation of the gradient is the most time-consuming operation. Yablonovitch et al. [44] developed a fast numerical implementation of this operation based on the adjoint method [45, 46]. This approach performs a forward simulation and an adjoint simulation, distinguished by the applied incident source. In the simple example of a density-based optimization task, with the FOM represented by the field intensity |**E**(**x**_0_)|^2^ measured at a certain diffraction order, the forward simulation calculates the electric field **E** ^fwd^*(**x**) at one grid point **x** under normal incident light. The adjoint simulation, on the other hand, calculates the electric field **E** ^adj^(**x**) under incident light from the target diffraction order. This formulation allows expressing the gradient with respect to the permittivity at the grid point ∂FOM/∂*ϵ*_*r*_(**x**) as [44]:

(1) ∂FOM∂ϵr(x)=Re{Efwd*(x)Eadj(x)}

with $E^{fwd*}\left(x\right)$ representing the complex conjugate of electric field from forward simulation. Equation (1) is rapidly evaluated by using only the local values of the electric field, and significantly speeds up calculations.

**Figure 2:**
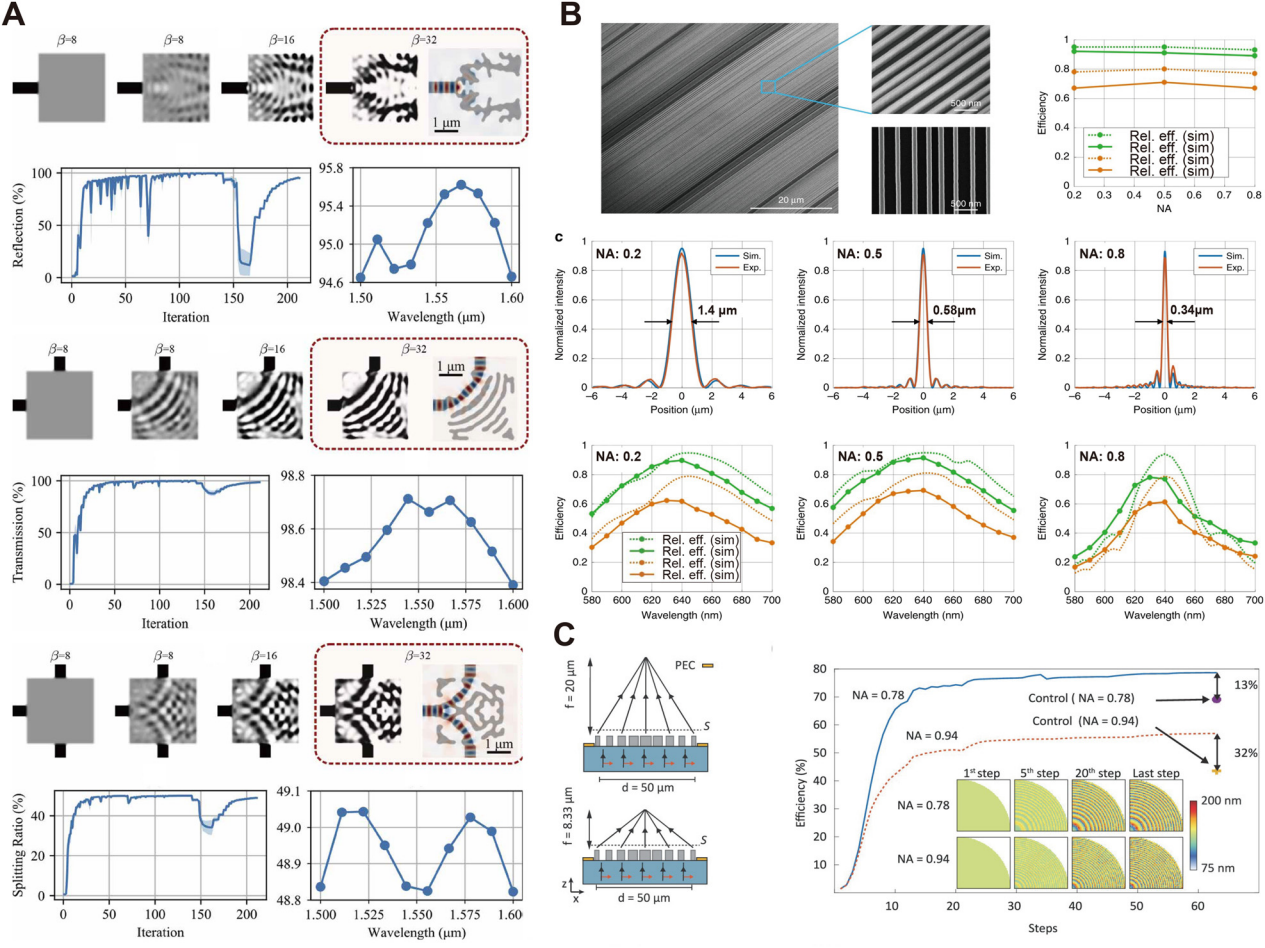
Topology optimization in nanophotonic inverse design (A) design-rule-constrained nanophotonic devices optimized with foundry design rule checks. The panels depict the optimization process and efficiency of top: mirror, middle: bend and bottom: T-splitter. Adapted from [33]. (B) High-NA, high-efficiency metalenses designed with linearized topology optimization. Top left: SEM images of the fabricated metalenses. Top right: experimental and simulated efficiencies of metalenses with NAs of 0.2, 0.5, and 0.8, respectively. Middle row: measured intensity at the focal planes of the designed metalenses. Bottom row: transmission efficiencies in the wavelength range 580–700 nm of the designed metalenses. Adapted from [31]. (C) Two dielectric metalenses with NA = 0.78 and 0.94, respectively (left) and the corresponding focusing efficiencies during the optimization process (right). Adapted from [34].

Figure 2A reports a group of broad-band photonic devices designed by density-based topology optimization. In the work [33], Hammond et al. also investigated foundry design-rule constraints, including both systematic limitations and fabrication errors. These are introduced as inequalities in the form of *g*_*k*_ ≤ *G*_*k*_. For each *k*, *G*_*k*_ is a positive constant indicating the constraint for a certain metric *g*_*k*_, including line width, line spacing, minimum area, and minimum enclosed area [47, 48]. To keep into account constraints of under and over-etching, which unavoidably occur at each fabrication step, Hammond et al. applied a conic image filter [49] on the design pattern. The modified design $\bar{\beta }$ satisfying all design-rule constraints listed above simulates the real-world fabrication scheme, including dilation and erosion. The topology design utilizes a classical binary representations of a silicon structure on SiO_2_ substrate. At each design point, the algorithm interpolates the permittivity as: $\epsilon_{r}\left(\bar{\beta }\right)=\epsilon_{\min}+\bar{\beta }\left(\epsilon_{\max}-\epsilon_{\min}\right)$ , where *ϵ*_min_ is the permittivity of SiO_2_ and *ϵ*_max_ is the permittivity of silicon. Constraining the parameter $\bar{\beta }$ in [0,1] allows continuously varying the permittivity across the design area and, during the initial stage of the optimization, it facilitates convergence by exploring all possible continuous distributions of design parameters. The optimization then introduces regularization terms that penalize all parameters that are different from zero or one, which does not represent silicon or SiO_2_. After convergence, the algorithm produces a discrete material distribution satisfying fabrication’s constraints. The above study contains three independent mirror, bend, and T-splitter designs working at 1500–1600 nm. The devices share a minimum line width of 90 nm, a minimum line spacing of 90 nm, a minimum area (for islands) of 0.08 μm^2^ and a minimum enclosed area (for holes) of 0.2 μm^2^. An increasing threshold coefficient that weights the penalization term ensures clear boundaries at the end of optimization. The algorithm iterates for 210 iterations and incorporates fabrication constraints during the last 70 iterations. Figure 2A shows the resulting devices, which achieve a simulated transmission efficiency above 94% within a processing time of 6–8 h. In 2020, Augenstein et al. [50] introduced mechanical loads into the optimization of nanophotonic devices, enabling additive manufacturing and further enriching the library of design-rule constraints.

Topology optimization reported also the implementation of metalenses at ultraviolet [51], visible [52, 53], infrared [54, 55], and millimeter [56] wavelengths with high numerical aperture (NA). Unlike conventional refractive lenses, metalenses focus incident light with sub-wavelength elements, which simulate the required surface curvature [57]. Researchers investigated non-periodic metalenses where the optical path varies among different designs to optimize the FOM, that is, the focusing efficiency at the focal length. Both relative (focused power/transmitted power) and absolute (focused power/incident power) efficiencies are accepted as part of the standard metric for metalenses. The optical properties, including deflection angle and phase response, are optimized at specific spatial points along the lens radius. As a result of this procedure, the numerical complexity of this class of designs is notably higher than the periodic metasurfaces mentioned above. To address this challenge, Phan et al. [31] studied a computationally efficient approach for the design of large-area metalens. The idea is to decompose the desired phase response along the radius into wavelength-scale segments and apply topology optimization to design isolated metasurfaces with scattering properties that linearly fit the corresponding segments. By integrating all discrete parts, the design converges to a functional metalens, with a time complexity that reduces from *O*(*L* ^2.4^) to *O*(*L*) for *L* being the degree of freedom in one-dimensional design space. Figure 2B depicts a series of 200 μm-wide metalenses designed to focus incident plane wave with NAs ranging from 0.2 to 0.8. The algorithm performs adjoint-based topology optimization on each 2 μm linear segment. Experimental results reveal efficiencies above 89% for all NAs in a wavelength range from 580 nm to 700 nm. The concept of linearization vastly reduces the system complexity compared to monolithic optimization. However, since the parallel tasks use perfectly matched layer (PML) boundary conditions [58, 59], the algorithm does not consider mode couplings between metasurface sections. This factor limits the linearization approach for an increasing number of segments, introducing strong couplings that finally obstruct convergence to an actual structure. Thus, the scheme searches for the best compromise between computational efficiency and linear fitting errors.

Recently, Mansouree et al. [34] reported an efficient design for high-NA metalenses with rectangular parameterization. Rather than directly modeling the permittivity at all design areas [60], the proposed method defines design variables as the widths of rectangular sub-wavelength structures. The design optimizes two metalenses with desired NAs of 0.78 and 0.94, formed by a total of *N* = 19,200 of 430 nm-thick amorphous Si bars. The nanostructures are symmetric in all four quadrants of the space, reducing the number of design variables to *N* = 4800. To calculate the gradient of the width variables, the authors implement an adjoint method with a perfect electric conductor (PEC) boundary condition. Figure 2C shows the resulting focal planes, located at 20 μm and 8.33 μm above the device surface for the metalens with NA = 0.78 and NA = 0.94, respectively. The rectangular parameterization inherits both advantages from periodic unit cells and monolithic design. It reduces the redundancy of bulk density variables while keeping the design diversity for complex, non-periodic nanostructures. The optimized metalenses with NAs of 0.78 and 0.94 achieve focusing efficiencies of 78% and 55%, respectively. This work assesses the performances of the proposed parameterization technique by designing two unit-cell metalenses as a control group under identical conditions. By comparing the simulated focusing efficiencies, the work reports a 10% performance improvement.

### 2.2 Heuristics and meta-heuristics

At variance with classical optimizers, heuristics and meta-heuristics exploit randomness in the searching process, imitating the behavior of different types of natural systems and phenomena. The primary example of heuristic methods is simulated annealing (SA), which originates in the context of non-differentiable, combinatorial problems, such as, e.g., the traveling salesman [61]. Problems of this type are mathematical ‘hard’ in the sense that the straightforward solution increases in time with a non-polynomial function, becoming quickly impossible to solve by any classical hardware.

Simulated annealing attempts to replicate the behavior of metals whose temperature decreases slowly, allowing the medium to converge towards a solid material, representing the global minimum of its potential energy. SA optimization imitates this process by setting the inverse design cost function *C*(***β***) as the potential energy of an equivalent molecular dynamics system, with each molecular particle described by a position *β*_*i*_ representing a design parameter that we intend to find. The annealing then proceeds to the global minimization of the particles’ positions ***β*** = [*β*_1_, …, *β*_*n*_] by progressively reducing the temperature with a suitably defined cooling scheme. If the temperature decreases sufficiently slowly, the probability distribution of the sequences of ***β*** found at each iteration follows the Boltzmann probability density $∼{\text{e}}^{-C\left(\beta \right)/T}$ of a classical thermodynamic system [62]. At *T* → 0, the molecular system converges to the set positions ***β*** representing the global minima of the cost function, solving the inverse design problem.

Zhao et al. [63] implemented this strategy to design diffusion metasurfaces that scatter the incident light uniformly in all directions. By exploring phase changing materials, this work designs a 2.4 μm × 2.4 μm reconfigurable metasurface [64] composed of 36 rectangular blocks as basic elements. The experimental results show a minimized directional reflection, with a 10 dB radar-cross-section (RCS) [65] reduction under normal incidence for both TE and TM polarizations. Another recent study by Xie et al. [66] proposed a magnetic resonator assembled by disordered nanostructures. The applied SA algorithm optimizes a five-by-five array of all-dielectric sub-wavelength scale resonators. The converged configuration reaches an experimentally measured peak magnetic field enhancement factor of 16.51 at 5.8675 GHz.

The main challenge of SA is that the convergence towards the optimal minimum lies in the heuristic idea of recreating a Boltzmann-type thermodynamic machine, which is not mathematically guaranteed to occur in every case. Ideally, addressing this problem requires sampling the probability space with different SA runs launched with diverse (random) input conditions. This operation, which could be time-consuming, has a drawback: it does not employ the knowledge acquired during the past since different SA runs proceed independently. Meta-heuristics schemes such as particle swarm optimization (PSO) provide a possible approach to address this issue. These optimizations take inspiration from the social behaviors of crowded biological systems, including ant colonies, fish, and birds flocks. Despite the apparent simplicity of their social interactions, these systems are highly efficient in exploring a given terrain when looking for food, flowers, and other equivalents ‘solutions’ to their search problem. In computational PSO, the system is initialized with sufficiently large swarms of randomly chosen *particles* ***β***_1_, …, ***β***_*n*_, with each ***β***_*i*_ representing a candidate solution to the problem. During each iteration, particles generate new configurations of ***β*** with the information from the best position found by each particle and neighboring particles. As in SA, the equivalent temperature of the system progressively decreases; unlike SA, however, temperature rescaling does not follow a pre-imposed annealing schedule but relies on the information acquired during the search [67].

**Figure 3:**
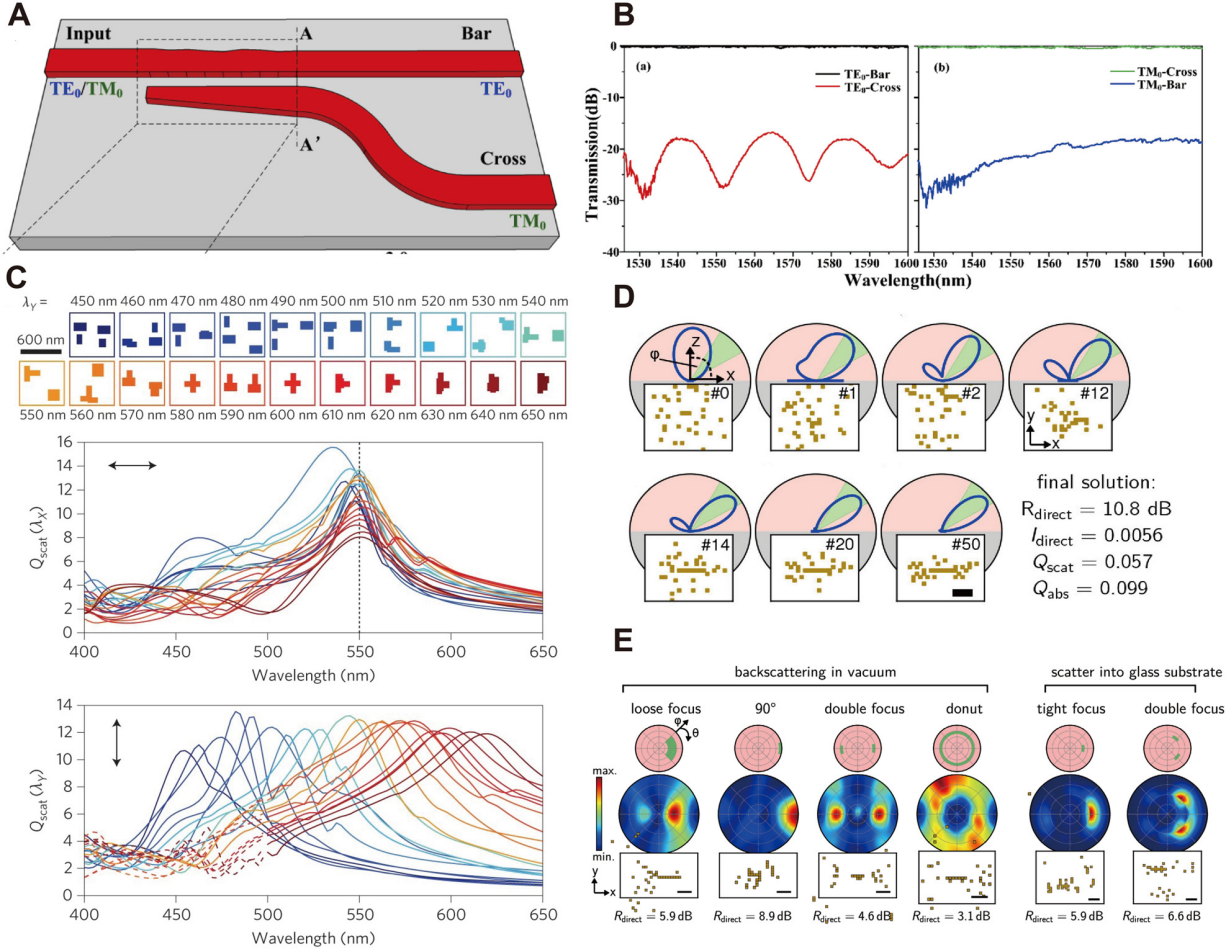
Heuristic optimization in nanophotonic inverse design (A) a silicon polarization beam splitter placed on SiO_2_ substrate. Bar and Cross are two polarization-related output channels. (B) The measured spectra of the fabricated beam splitters in (A). (A) and (B) are adapted from [68]. (C) Multi-resonant dielectric nano-antennas with fixed resonance at 550 nm for one polarization X, and ranging resonance peak from 400 nm to 650 nm for the other polarization Y. Adapted from [76]. (D) Plasmonic gold antennas with maximum scattering on the desired direction. Each panel shows the fittest solution at different iterations, starting from initialization to convergence. (E) Optimized examples of directional antennas for various target angels and scattering environments. Left: backscattering in vacuum. Right: scattering towards glass substrate. (D) and (E) are adapted from [78].

Chen et al. [68] reported recent progress on PSO designed polarization beam splitters. The devices strengthen its compatibility to complementary metal-oxide-semiconductor (CMOS) by optimizing the silicon-on-insulator (SOI) structures. As shown in Figure 3A, the dashed area constrains the design space, which completes the functionality as a counter-tapered coupler. To reduce computational complexity, the authors split the coupler into ten silicon stripes within a total coupling length of 5 μm. The algorithm includes particles with positional vectors *ps*_*n*_ represented by widths of the segmented stripes and velocity vectors *ve*_*n*_ employed to update the former. The FOM is defined based on the polarization extinction ratio between output channels. As TE and TM waves are separated into the Bar and Cross port, respectively, the FOM is calculated by *P*_TE_Bar_/*P*_TE_Cross_ + *P*_TM_Cross_/*P*_TM_Bar_, where *P* represents the transmitted power. After initialization and EM simulation, each particle records its best position *bp*_*n*_ and the global best position *gp*_*n*_ among the swarm. The update formula for *ps*_*n*_ and *ve*_*n*_ is as follows:

(2) $$\begin{array}{cc} ve_{n+1} & =w_{\text{I}}\times ve_{n}+r_{1}\times \mathrm{rand}\left(\right)\times \left(bp_{n}-ps_{n}\right)\\ & \;+r_{2}\times \mathrm{rand}\left(\right)\times \left(gp_{n}-ps_{n}\right)\\ ps_{n+1} & =ps_{n}+ve_{n} \end{array}$$

where *w*_I_ is the inertial weight, describing the momentum of previous movements. 0 ≤ *r*_1_ ≤ 1 and 0 ≤ *r*_2_ ≤ 1 are the cognitive and social rate, representing the weights of individual *bp*_*n*_ and social memory *gp*_*n*_ of past best configurations. Figure 3B shows experimental results revealing high polarization extinction ratios over 16 dB for both TE-type and TM-type grating couplers obtained with PSO. Recent PSO-based researches also provide solutions across a wide range of nanophotonic devices including power splitters [69], solar cells [70], achromatic metalenses [71] and phase changing antennas [72].

The main issue in PSO is the speed of the algorithm’s convergence, which follows the set of worst-performing particles. Evolutionary schemes, such as, e.g., genetic algorithms, try to address this issue by selectively mixing individual information arising from the design population [73]. Spuhler et al. [74] first proposed a genetic algorithm for the design of fiber-waveguide coupler. By splitting the SiO_2_/SiON functional area into segments with identical lengths and flexible widths, a population of design candidates containing a distinct width sequence evolves with random mutations, cross-overs, and die-out. A non-intuitive structure differing from any existing design emerges after 1132 optimization steps, with a 2 dB reduction on coupling loss compared to direct butt-coupling devices.

Following this initial genetic approach, a series of photonic devices have been proposed with increasing design complexity during the past decade [75]. Figure 3C reports a cluster of dielectric nano-antennas for polarization-encoded color display obtained by genetic algorithms. The work exploits sub-wavelength nanostructures that alter their optical properties for different light polarizations. To address this design task, Wiecha et al. [76] proposed an evolutionary multi-objective optimization approach, which maximizes simultaneously the scattering efficiency at demanded wavelength, as well as both TE and TM polarizations. The periodic unit cell is a set of four silicon blocks, with horizontal sizes ranging from 60 nm to 160 nm. The entire design space is a 600 nm × 600 nm area, with available parameter combinations larger than 1 × 10^15^. The algorithm defines a fitness function that integrates polarization-dependent metrics and evaluates all candidate designs in each iteration. The selected high-performance designs reproduce a new generation by cross-over and random mutation and finally lead to a convergence indicated by the Pareto front, a standard metric for multi-object programming [77]. In this specific task, the Pareto front is the set of design parameters whose modifications enhancing the scattering at one polarization cause the efficiency to drop for the other polarization. The Pareto front keeps expanding as the optimization processes until the device reaches saturation, indicating the best performance. As depicted in the bottom panel of Figure 3C, the X-polarization (TE or TM) scattering peak is at 550 nm. In contrast, the Y-polarization (TM or TE, respectively) scattering peak is evolved gradually from 400 nm to 650 nm, all with consistent efficiency (within ±20%) in both polarizations. Compared to direct search, the evolutionary algorithm rapidly explored the design space within 100 iterations from a population of 20 candidate designs.

The work in [78] exemplifies a light-directional antenna, which maximizes the ratio of direct scattering at a specific direction to the residual scattering directions. Inheriting similar optimization strategies from [76], the authors leveraged a binary-shaped design space containing 40 nm × 40 nm × 40 nm gold blocks (represented by “1”) and air (represented by “0”). The total amount of all possible arrangements extends to 10^111^. As shown in Figure 3D, the algorithm starts with an initial population size *N*_p_ = 500 and reaches convergence after 50 generations for fixed azimuthal angle *φ* = 45°. The scattering antenna evolves spontaneously to form a structure resembling a Yagi–Uda RF antenna [79] with one driving feed, one reflector, and one director. Figure 3E reports examples of scattering antennas optimized under different conditions (e.g., propagation media, diffraction windows) from this work.

## 3 Inverse design by deep learning

Recent trends in inverse design take advantage of the large body of research available in the context of deep learning [80]. Deep learning offers advantages over classical optimization schemes, such as the ability to predict design output given a set of design parameters at the input, without the need of iteratively minimizing the FOM function. Deep learning encompasses a large variety of schemes and diverse taxonomies of classification. Traditionally, a standard classification divides deep learning into supervised, unsupervised, and reinforcement learning schemes. Supervised learning [80] uses labeled datasets to train the network to learn the required input–output tasks, declaring the demand for high-quality, large-scale datasets. This condition is relaxed in unsupervised schemes [81], which do not use any pre-assigned information. Reinforcement learning, on the contrary, trains an automatic agent to control the design variables, with possible actions such as increasing/decreasing thickness, changing material types, increasing/decreasing unit diameters [82].

Due to the increased complexity of nanophotonic devices, modern inverse design schemes often integrate these different learning models into a single approach [83]. Therefore, state-of-the-art inverse design techniques have blurred boundaries between these three learning domains. Another possible classification follows the recent progress in the computer vision community [84], which discusses deep learning schemes in terms of discriminative and generative models. In the specific context of nanophotonic inverse design, discriminative models learn the one-to-one mapping between optical responses and material configuration layouts. Generative models, on the contrary, learn the statistical distribution of potential designs that minimize the FOM, achieving a one-to-many mapping, i.e., a single desired output and many possible resulting designs.

### 3.1 Discriminative models

Discriminative models bypass the repeated EM input–output simulations, often the bottlenecks of the optimization schemes reviewed in the previous section. Discriminative models are essentially unique configurations of deep learning architectures that learn the relationship between designs in the parameter space and their optical responses (reflection, transmission, amplitude or phase) [85–91]. Based on the study of plasmonic nanostructures, Malkiel et al. [92] introduced a bidirectional network composed of geometry-predicting-network (GPN) and spectra-predicting-network (SPN). The authors train the GPN in a supervised manner with a dataset composed of 15,000 randomly generated device layouts containing eight geometrical parameters, including width, height, rotation angle, and the presence of positional elements in the H-shaped framework. Additional to the device data, finite elements simulations (FEM) calculate the corresponding transmission spectra. The GPN predicts the geometrical design parameters by processing 86 sample points from both TE and TM spectra and dispersive material coefficients. After training, the SPN, constructed in a cascade with the GPN output layer, retrieves the spectra from the eight geometrical parameters launched at the input. The network efficiently leverages the limited dataset by using unseen shapes generated by GPN as the training set of SPN. The loss function and gradient propagate to each layer in both networks. The end-to-end training of the network converges within 2 h, after which the time required to complete queries for potential nanostructures lies in the millisecond range. As exemplified in this work, the GPN retrieves classic nanophotonic structures such as nanobars, L-shaped, and split-ring resonators. To efficiently extract the hidden features from nanostructures, the training of a forward model (device-to-response) and an inverse model (response-to-device) is beneficial. Similar to the GPN and SPN introduced above, the two models can be either constructed and trained independently [93] or with inner connections, such as the generative models that will be introduced in the next section.

**Figure 4:**
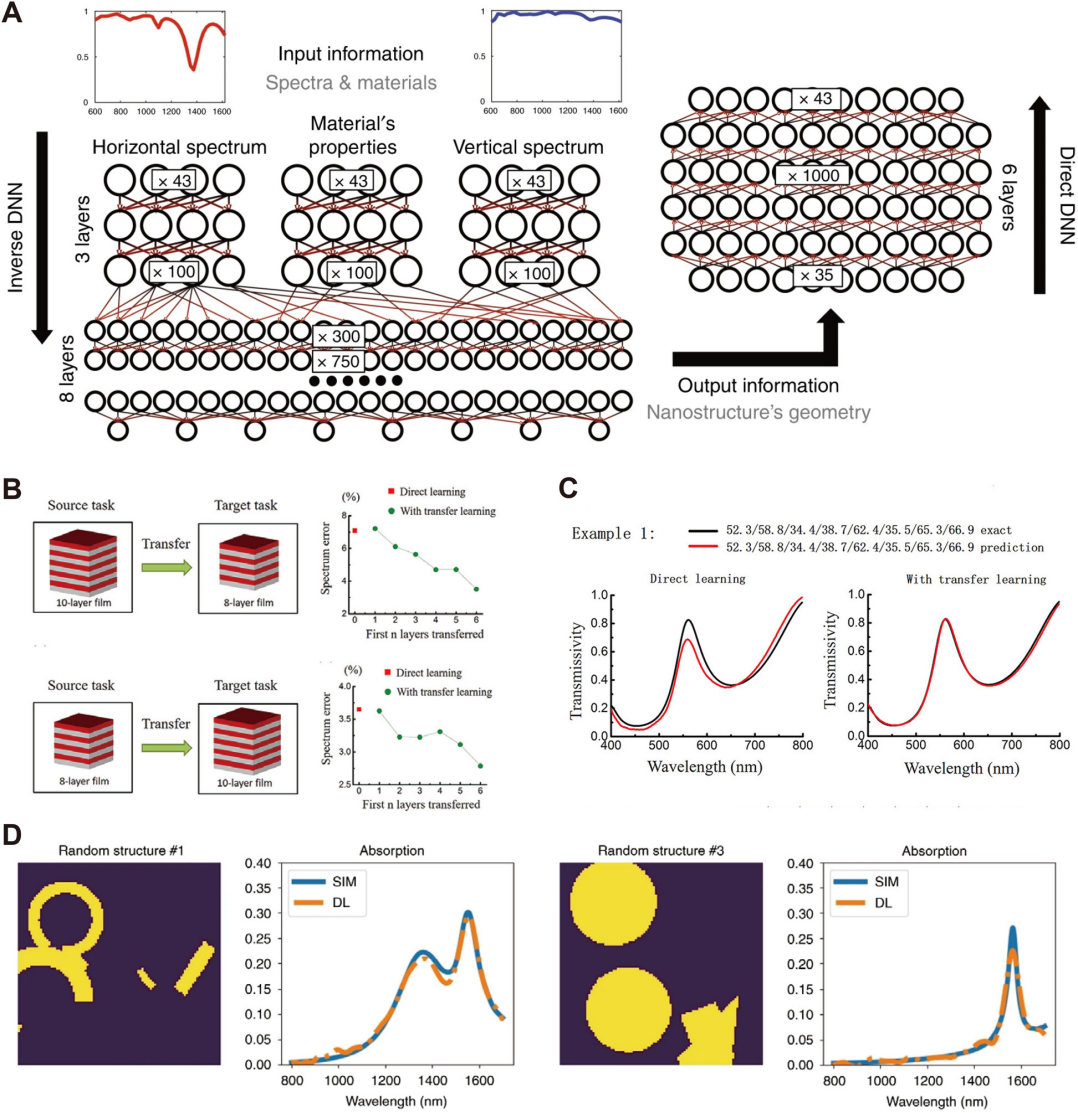
Discriminative deep learning model for spectra predicting and design parameter retrieval (A) bidirectional network for plasmonic nanostructure design. Left: geometry predicting network with TE&TM spectra and material properties as input. Right: spectra predicting network, concatenated to the first network in a cascaded structure. Adapted from [92]. (B) Knowledge transfer between the design of 8-layered and 10-layered transmissive films. As the shared network layers keep increasing, both transfer schemes (8-layered to 10-layered and the inverse) show better prediction precision compared with direct training. (C) Example of predicted spectra compared to real spectra from the 8-to-10-layer transfer learning tasks. Left: direct learning. Right: transfer learning. (B) and (C) are adapted from [89]. (D) Results of the discriminative design on the plasmonic silver absorber. The device structure is encoded as 2D images, with absorption spectra predicted by the RNN and FCN layers. Adapted from [88].

Other discriminative models leverage the generalization ability of NN in two main directions [94, 95]. On the one hand, they exploit the fact that a trained system predicts novel results for an arbitrarily large number of queries; on the other, they use the information acquired by the NN during training and transplant it via transfer learning [96] in a different task. Work proposed by Qu et al. [89] embedded and demonstrated transfer learning in a nanophotonic inverse design scheme. The approach includes the precise prediction of transmission spectra for an 8-layer thin film and a 10-layer thin film. Figure 4B shows an overall schematic of the transfer learning process. A 7-layer fully connected network (FCN), acting as a base network, is trained on a complete dataset containing 50,000 simulation examples. After the training, a second FCN acting as the transferred network replaces the top *n* layers of the base network, leaving the rest 7-*n* layers sharing weights from the trained one. The second network demonstrates the advantage of transfer learning by training 500 examples sliced from simulation results in a different design task, which are insufficient for training a model from scratch. As a quantitative example, Figure 4B illustrates the transfer learning schemes where the source task and target task switch between 8-layer and 10-layer films. By increasing the number of shared layers, the mean square error (MSE) reduces by 50.5% and by 23.7% in the 8-to-10-layer and 10-to-8-layer transfer model, respectively. The improved performance reveals that the knowledge from the complete dataset is preserved and leveraged in training the sliced dataset. Figure 4C shows a test example, comparing the performance of direct learning strategy and transfer learning in a working bandwidth between 400 nm and 800 nm. This work demonstrates that while comprehensive modeling of physical effects could be difficult to achieve, the implicit knowledge carried out by pre-trained NN is a practical approach to mitigate this issue, especially when simulations are time-expensive.

The examples discussed above include different FCNs to grasp the physical relation between characteristic parameters of a design and the output spectral response furnished by the system. A recent trend of nanophotonic inverse design [97] introduces a high-level hierarchical representation of data for fast image processing and sequential analysis. Sajedian et al. [88] exemplified a design of periodic silver nanoparticles based on a combination of convolutional neural networks (CNN) and recurrent neural networks (RNN). Two-dimensional images of 100 × 100 pixels 2D encode the top view of three-dimensional structures with a definite constant thickness along the *Z*-axis. The authors use a training dataset with random images comprising a large variety of geometries, width, length, position, and rotation. Finite-difference time-domain (FDTD) simulations [98] computes the absorption spectra of 100,000 structures in the dataset. The applied CNN is ResNet [99], which is a powerful architecture in computer vision for pattern recognition problems. ResNet contains shortcut connections that link non-adjacent network layers, providing a straight path for identity mapping [100]. This feature prevents the gradient vanishing and gradient exploding issue in training deep CNNs and grants the model a more robust capability to extract information from the 2D structure.

The output arising from ResNet follows a time-distributed RNN layer connected to a final predictive FCN stage. Due to the increased complexity of this network, the reported training requires approximately one week to converge. The trained network predicts the absorption spectra of silver structures. As a proof of concept, the authors test the model on a validation dataset containing 1000 sample points on the spectra between 800 nm and 100 nm, which reaches an MSE = 4.259 1 × 10^−5^. As shown in Figure 4D, vital characteristics such as peaks and valleys are fitted well with the simulation results. This work extends the design space from geometrical parameters to arbitrary 2D shapes by applying image processing networks.

An empirical principle in machine learning dictates that the higher the NN model complexity, the larger the dataset needed to obtain good prediction accuracy on untrained data [101]. This condition also applies to and challenges NN-based inverse nanophotonic design. To train a more sophisticated NN with linearly expanding layers, the size of datasets can increase drastically, requiring vast amounts of EM simulations. From this point of view, the computational cost of training deep NN scales up by requiring more resources than optimization-based methods. However, the main strength of the deep learning-based inverse design is transfer learning: transferring the knowledge acquired by the progressive solution of many numerical simulations into different tasks. In the future of NN-based inverse design with increased model complexity, we expect a more decisive role of transfer learning techniques in formulating the network model.

### 3.2 Generative models

An issue with discriminative models is that one trained network can only provide a single, one-to-one mapping between the design variables and the spectral response at the output. However, in optics, various distinct structures can achieve a specific response. Generative models address this issue by computing distributions of possible structures that achieve analogous target responses. The models start the training by sampling a ‘latent space’ constructed from known distributions of random vectors. In most cases, these probabilities are multi-dimensional Gaussian distributions, with enough randomness provided for the model to learn a complex projection from the latent space to the distribution of functional nanophotonic devices. In general, the generative models decode the sampled vector to produce design parameters. Different generative models implement diverse strategies for learning these complex projections.

**Figure 5:**
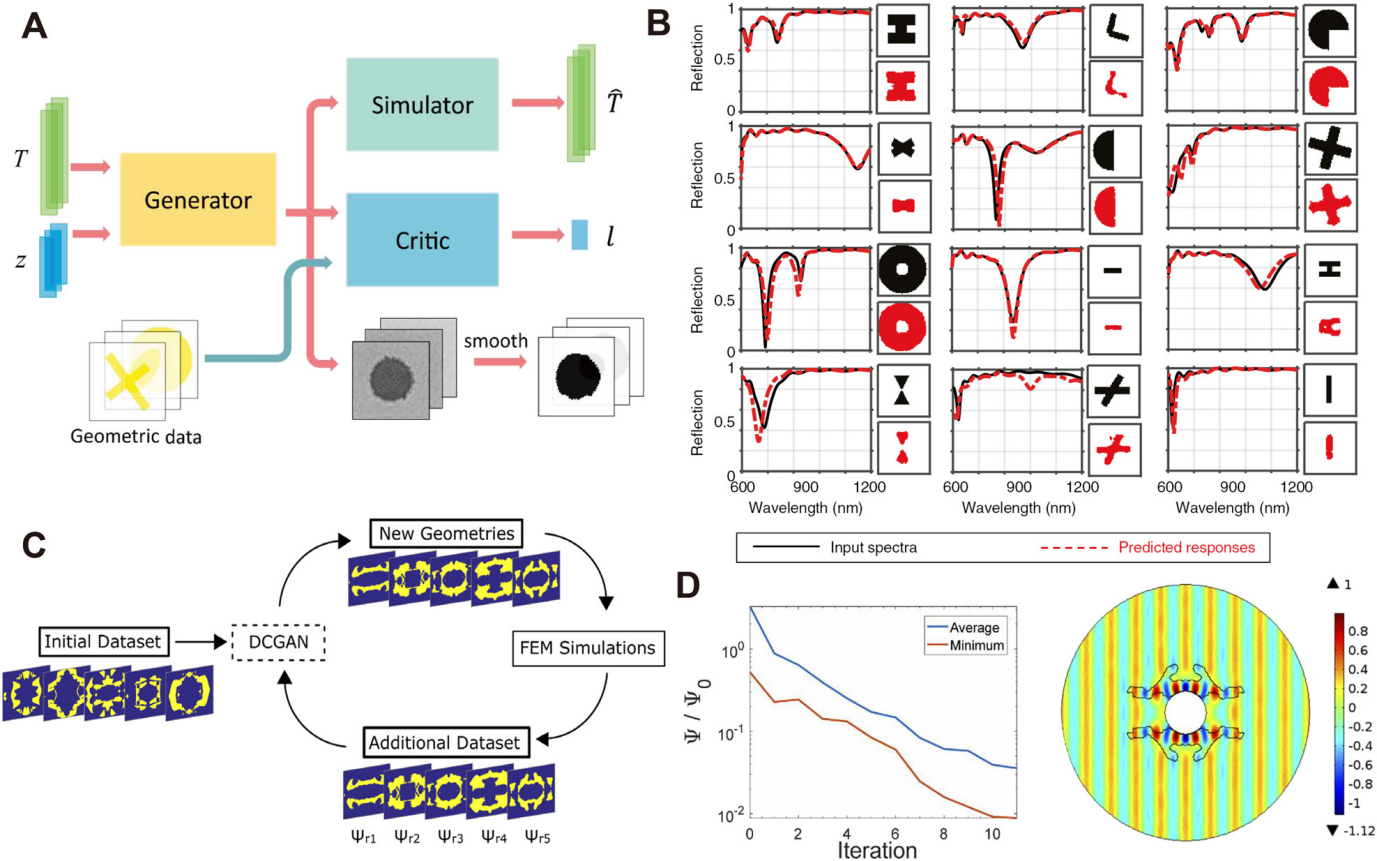
Generative adversiral network (GAN) implemented in nanopotonic inverse design (A) GAN’s generic schematic applied in nanophotonic design, which includes a generator, discriminator(also known as critic), and simulator. The generated device pattern is further optimized for fabrication by a smoothing procedure. Adapted from [108]. (B) A GAN-based silver structure design under arbitrary reflection spectra. The panel shows nine examples of device binary images generated by GAN and the corresponding reflection spectra as condition (solid line) and predicted response (dash line). Adapted from [110]. (C) Basic training strategy of GAN in the design of optical cloaks. The panel shows a successive data augmentation technique, where FEM simulations evaluate the newly generated geometries to complement the initial dataset. (D) Left: average and minimal scattering coefficient among the top-1000 configurations generated by the network. Right: the scattered field in an optimized 2-dimensional optical cloak. (C) and (D) are adapted from [112].

Generative adversarial networks (GANs) [102] represent the most prominent class of deep learning generative methods developed during the past decade in various research fields, including image translation [103, 104], privacy protection [105], natural language processing [106]. We here summarize a generic schematic of conditional GAN (cGAN) [107] architecture implemented in nanophotonic inverse designs. The essential parts of a GAN comprise a generator **G** network, which provides the design, and a discriminator **D**, also known as the critic that evaluates the provided designs. In the context of inverse design, an additional simulator network **S** serves as an external unit for predicting the optical response from the generated design and measuring the pre-defined FOM corresponding to specific tasks. The models train the generator **G** and discriminator **D**, simultaneously and adversarially. As depicted in Figure 5A, the pioneering work of Liu et al. [108] defines **z** as the random vector sampled from latent space, and **T** as the conditional variables that indicate the target response, which is the transmission spectra measured across TE and TM polarizations in this work. Under the goal of modeling and designing photonic devices, the generator **G** produces structural designs **G(*z*, *T*)** resembling actual structures **X** in the provided dataset. In contrast, the discriminator **D** is trained to distinguish **G(*z*, *T*)** from **X**. To carry out this task, the discriminator **D** predicts a [0,1] ranged value *l*, which represents the probability of each structure being real (existing in the dataset) or fake (generated by **G**). The authors train the discriminator **D** to minimize the distance between *l* and the actual categories, while the objective of the generator **G** is to maximize this distance. These adversarial training goals finally converge to a Nash equilibrium [109], representing a balance between the productivity of **G** and the discriminative ability of **D**. At this stage, the generated design images **G(*z*, *T*)** share similar geometrical features with **X** in the training set, while **D** fails to identify them from the actual structures **X**. According to various design tasks, the simulator **S** is pre-trained on labeled datasets, including device structures and the corresponding simulation results (transmission/reflection/absorption spectra, scattering coefficient) in different polarizations and wavelength ranges. It calculates the FOM by predicting the optical response on the generated structure **G(*z*, *T*)**. The algorithm then integrates the optimization of FOM into backpropagation and gradient descent of **G** layers. The generator can produce optimized designs at convergence with all necessary structural constraints and topologies shared in the dataset.

Rho et al. [110] proposed an alternative deep cGAN model for predicting reflection spectra of silver nano-antennas. The cross-sectional design space of the surface reflectors is limited in a square of 500 nm × 500 nm, represented by 64 × 64 binary images. FDTD simulations provide reflection spectra with 200 frequency points sampled from 250–500 THz. This work further optimizes the generator network architecture by constructing two separate convolutional blocks for the feature extraction of the random vector **z** and conditional variables, specifically, user-defined reflection spectra. Then the model fuses the output of two convolutional blocks into one 1024-channel tensor and eventually produces the design image. Instead of building an independent simulator model shown in Figure 5A, the FOM is measured directly from FDTD simulation on generator-suggested designs. In the early stage of training, the generator produces chaotic distributions of pixel values. As the training proceeds, the output images gradually show precise geometric shapes without additional regularization. Figure 5B shows examples of generated designs. The solid black spectra are the design objectives corresponding to the dataset’s structural images (black). Moreover, the structures drawn in red are the GAN-optimized designs, which share a similar topology yet comprise deformations such as erosion, dilation, and mirroring. The mean absolute error (MAE) between the reconstructed reflection spectra (red dash line) and simulated spectra is 0.0322. This result reveals GAN’s strength in modeling one-to-many mappings. Furthermore, the authors test the model on user-defined reflection spectra generated by curve functions. Despite the non-existing nature of such reflection patterns, the model achieves a <5% MAE between the reconstructed and the user-defined spectra, revealing a good approximation. An advantage of GANs is to explore the latent space implicitly, with seed vectors sampled from pre-defined probability distributions [111]. The generated randomness helps the networks find equivalent structures that differ from the training set, further enriching the existing dataset. Recently, Blanchard et al. [112] proposed a successive training strategy employing GAN-based data augmentation for the design of dielectric optical cloaks. The design space is binary images of a split ring resonator (SRR) with a central PEC circular boundary. When the incident light propagates through the cloak, the wavefront remains identical. This work defines a relative scattering coefficient to measure the difference between background field and scattered field. Similar to the architecture described in Figure 5A, the proposed approach comprises a generator, a discriminator, and a forward simulator that is pre-trained to predict scalar scattering coefficients. The authors freeze the weights of the forward network during GAN training while its output participates in the backpropagation. Minimizing the relative scattering coefficient demands the forward loss to be calculated simply by the distance between the predicted scalar value and constant 0. A total number of 13,000 FEM simulation results initialized the dataset, where the cloaking performance varies according to the shell geometries.

Figure 5C illustrates the unsupervised data augmentation technique applied every 60 iterations, where the forward simulator first evaluates newly generated configurations. The 1000 best geometric structures with the lowest scattering coefficients are simulated using FEM and added to the dataset. The model then utilizes the updated dataset to refine the prediction accuracy of the forward simulator and subsequently train the GAN. As a consequence of such a joint training strategy, GAN-generated geometries converge to a stage with minimum scattering coefficients by backpropagation, with high-quality samples increasingly dominating the dataset. Figure 5D exemplifies the learning curve of the average and the best scattering suppression at every 60 epochs. The right panel visualizes the normalized H_z_ field of the optimal configuration, on which the ratio of the scattered field to background field reaches 0.0089, implying almost uniform propagation.

**Figure 6:**
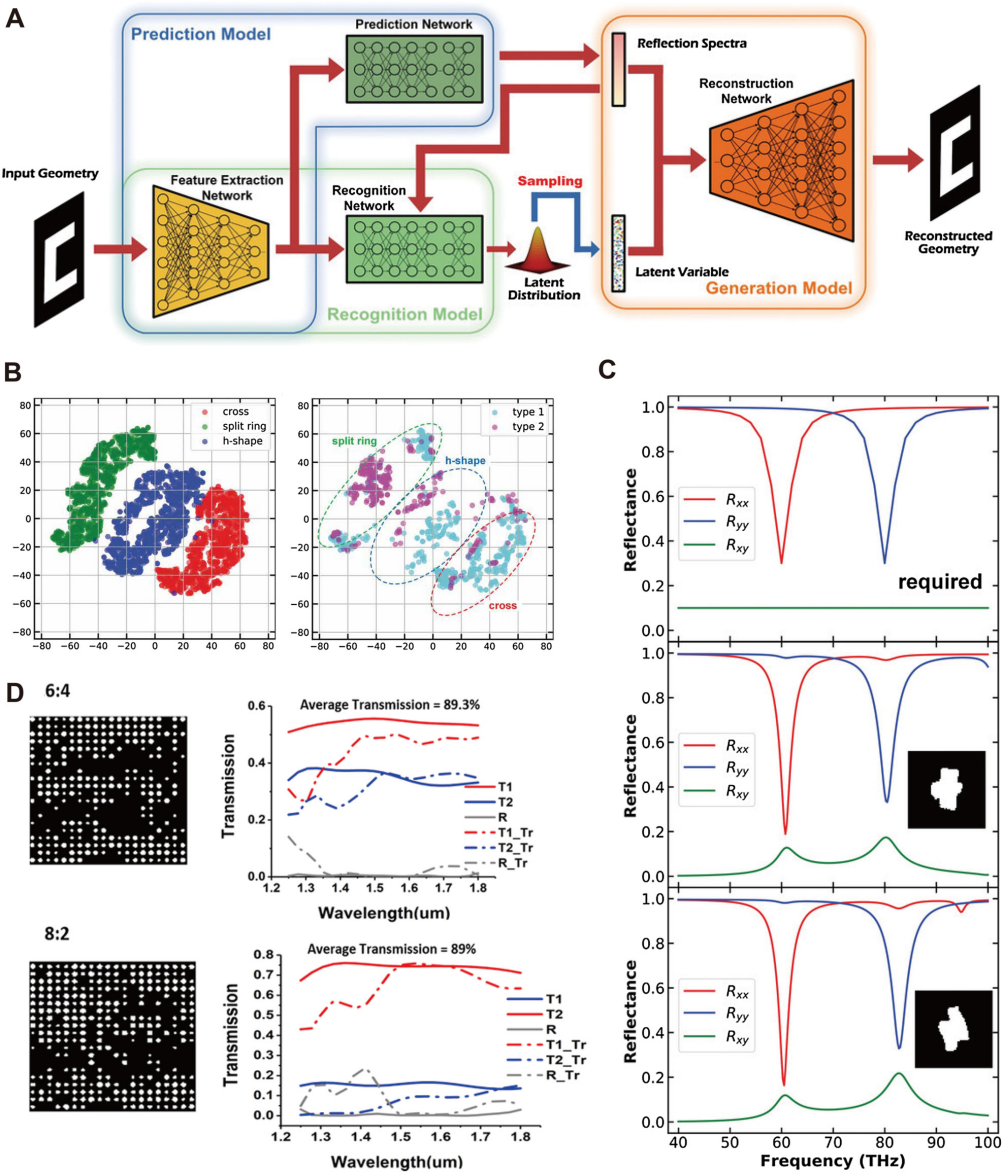
Variational autoencoder (VAE) assisted nanopotonic design (A) a c-VAE based metamaterial design system, including a prediction model, recognition model, and generation model. (B) Visualization of the reduced latent space, where three types of geometries and two-types of optical responses are depicted on the plot. (C) Examples of on-demand metamaterial reflector design. Two equivalent structures are generated under the given spectra condition. (A), (B) and (C) are adapted from [113]. (D) Examples of broadband power splitters with 2.25 × 2.25 μm^2^ footprint. A 550 nm working bandwidth is achieved for all devices with arbitrary splitting ratios. Adapted from [116].

Ma et al. [113] introduced the concept of conditional variational autoencoder (cVAE) [114, 115] in the inverse design of reflective metasurfaces. Depicted in Figure 6A, the cVAE contains three correlated sub-models, respectively, recognition model, generation model, and prediction model. The authors train the prediction model on simulated data. After convergence, the model can predict reflection spectra that cycle into the other two models as additional information. Acting as the ‘encoder’, the recognition model projects the input geometry to a 20-dimensional space by assigning mean value *μ* and variance *σ* to the pre-defined Gaussian distribution. A re-parameterization technique is applied to sample latent vectors from this probability density function, described as follows:

(3) z=σi+μ

where **i** follows a standard Gaussian distribution. The latent vector **z** is then decoded by the generator model to reconstruct the input geometry, given the reflection spectra as conditional variables similarly to the GAN training scheme mentioned above. The training proceeds to show the formation of a clear unit cell pattern with accurate optical response in the range 40–100 Thz. VAE is a highly structured model, where adjacent points in the latent space correspond to continuous change on device structures. Exploring the converged Gaussian distribution reveals three groups of devices in the scatter plot of Figure 6B, respectively, cross, split-ring, and H-shaped nanostructures. The authors further characterize the reflection properties by locating the most robust resonance and dividing the nanostructures into two types with resonance frequency under and above 60 THz. As depicted in the right panel of Figure 6B, different types of spectra distribute among all clusters. This result demonstrates that prior-defined device form factors can be exploited in the design while each form sufficiently covers a wide range of optical responses. Figure 6C shows an example of generated structures under given conditions, which include TE to TE (*R*_*xx*_), TM to TM (*R*_*yy*_), and TE to TM (*R*_*xy*_) reflection spectra. The required response contains two resonance valleys on *R*_*xx*_ and *R*_*yy*_, respectively. Two generated structures show in the below panels, where their numerical simulations show major agreement with the required spectra at resonant points.

In the second example of cVAE-assisted inverse design, Tang et al. [116] developed a set of nano-patterned power splitters with user-defined split ratios. The devices are silicon-based square couplers with a footprint of 2.25 μm × 2.25 μm, which distribute 400 independent etching hole positions across the square area. The configurations of etching holes on the coupler is a 20 × 20 vector scaled in the range [0,1], each variable indicating the absence (<0.3) or a rescaled diameter size (>0.3) of one etching hole. The dataset contains 15,000 simulated transmittances of randomly generated hole vectors. Two ideally flat transmission spectra constitute the dependent variables to achieve the desired split ratio of the output ports. Above the original c-VAE architecture with a latent space, the authors introduced the adversarial block [117], an alternative branch of NN that validates the sampled latent vector **z**. This network estimates a diversity of optical responses from points across the latent space distribution, following the fact that the actual response of a power splitter is commonly irregular and not smooth as the ideal condition. The network obtains the highest performance by forcing the latent space distribution to generate various yet effective samples that correspond to the defined split ratio. Figure 6D exemplified two prototypes generated by the model, with splitting ratios of 6:4 and 8:2, respectively. The optimized design achieved over 87% total transmission efficiency across the working bandwidth from 1250 nm to 1800 nm. The training in [116] also implements an active learning method, where data augmentation based on FDTD simulations operates after each stage of training. Besides VAE, other autoencoder-like models exclude the random sampling process by directly reducing the input dimensionality to a fixed-length hidden vector. This lightweight model also accelerates nanophotonic applications with low computational burden, such as the design of reflective metasurface [118] and photonic topological insulators [119].

Illustrated in the above examples, the main advantage of generative versus discriminative models is producing novel data. Generative models learn the representation of the data structure and can supplement and refine the training set. Among various application fields such as text generation [120], texture filling [121], and image translation [104], the representation-learning of generative models also show significant improvements for inverse design tasks. Initially trained on a dataset containing nanostructures and optical properties, the converged network produces an optimized design distribution given certain conditions. The typical approach of external validation is EM simulation, which labels the generated design with the corresponding spectral response and eventually contributes to enhancing the original dataset. Besides this, direct experimental measurement on the fabricated devices also serves as an effective data generation method, compared to the intense computational demand of numerical simulations.

## 4 Merging deep learning with optimization techniques

While deep learning models benefit from strong predicting abilities, they often require vast initial training resources. Efficient training of NN to learn to express user-defined functions is presently still a subject of intense research. Even with the latest generation of training schemes, instabilities and non-convergence problems [122] persist, especially when the network architecture becomes sufficiently large [123]. On the contrary, optimization-based inverse design requires the knowledge of a single resource, the FOM, to tackle multiple design objectives and independent tasks at one time. Optimization schemes provide a more straightforward yet solid evolution framework for the design parameters. Blending deep learning and optimization methods is a current trend in nanophotonic inverse design. Such a hybrid framework leverages fast gradient retrievals and low-cost evolution from optimization while inheriting the generalization ability of deep learning models to reduce the design dimensionality and accelerate convergence towards the optimal design.

**Figure 7:**
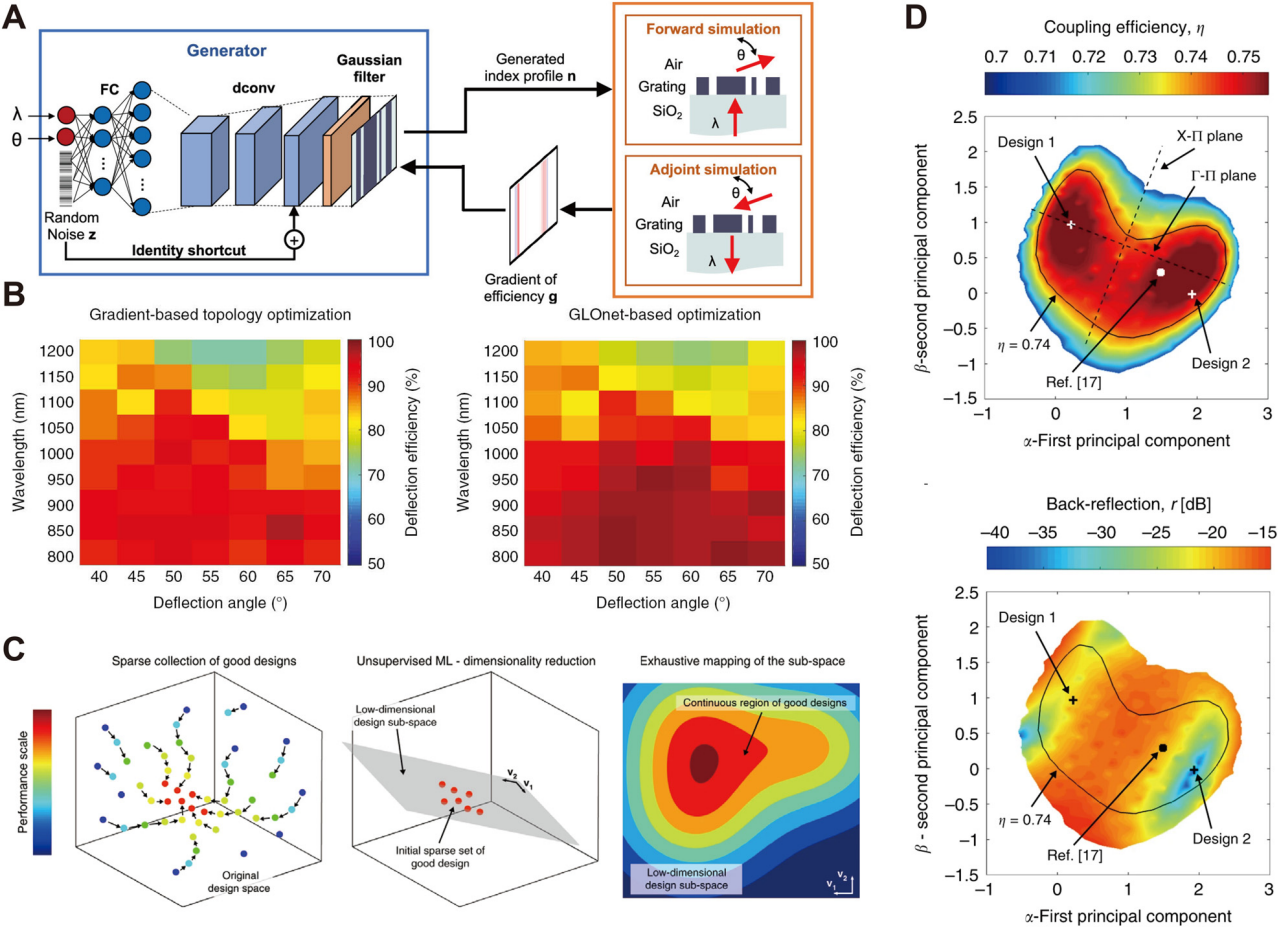
Hybrid design methods with deep learning models performing complex projection on design space (A) schematic of global optimization network (GLOnet) as a combination of GAN architecture and topology optimization approaches. The generated design candidates are utilized to calculate the gradient for the FOM by adjoint methods. Adapted from [15]. (B) Deflection efficiency of devices optimized by gradient topology optimization and the proposed GLOnet-based approach. The working wavelength varies from 800 nm to 1200 nm and the deflection angle varies from 40° to 70°. Adapted from [124]. (C) The PCA-based dimensionality reduction of design space for vertical grating couplers. Exhaustive mapping is conducted at the reduced sub-space to generate the optimized design. (D) Top: coupling efficiency of the optimized devices measured from the principle hyperplane. Bottom: back-reflection measured from the principle hyperplane. (C) and (D) are adapted from [16].

Figure 7A shows a hybrid design model based on GAN and topology optimization. In this work, Jiang et al. [15] demonstrated that the adjoint method could reduce the computational cost for training deep NN. The proposed model, named GLOnet, replaces the discriminator in standard GAN architectures with EM simulations. The design divides the diffractive metagratings equally into 256 segments, of which a 256-dimensional vector represents the refractive indexes. The desired deflection angle and working wavelength are the conditional variables to the generator. GLOnet implements an image processing model concatenated with a Gaussian filter to eliminate small discrete features on the structure to fulfill fabrication constraints. As described in Section 2, the gradient of FOM is directly calculated from the forward and adjoint simulation results, while all network layers backpropagate this gradient to update weights. The proposed method combines fast gradient calculations from topology optimization with the generation capability of GAN. This work has no dependence on the initial dataset, as all generated patterns are simulated dynamically at each iteration. Like topology optimization, GLOnet introduces an additional penalization term to discretize the device structure by forcing the refractive indexes to approach one of the two given materials (silicon or air). An increasing trend of design structures with higher transmission efficiency and binarization of refractive indexes emerge during the training.

In the following work [124], the authors further compared GLOnet with pure topology optimization methods. Figure 7B visualizes the optimized device performances under working wavelength varying from 800 nm to 1200 nm and deflection angle from 40° to 70°. The results reveal a performance improvement that 90% of the GAN-generated devices achieve higher efficiency than topology-optimized ones. The authors explain this performance by considering that GLOnet searches the entire design space via deep NN, while topology optimization performs a one-path search for every independent task. Crosstalk among different GAN-generated samples accelerates the global convergence by exploring possible high-quality devices, granting them a higher impact factor in the final probability density. A following up work [125] expands the model to a multi-objective flat design where the network produces both material types and device layouts.

In the previous examples, deep NN architectures, including GAN and VAE, model the design space. Daniele et al. [16] proposed an alternative path supported by principal component analysis (PCA) in the design of vertical grating couplers. Five geometrical parameters constitute the dimensions of the design space. An effective Fourier-type eigenmode expansion simulator [126] calculates the coupling efficiency from the device to a vertically placed optical fiber. An auxiliary NN predictor is trained on this dataset to accelerate the first-stage optimization. As shown in Figure 7C, the initial optimization conducted by a local search algorithm generates a sparse collection of rough designs, whose proximity in the design space represents the potential manifold of high-performance devices. Then, PCA reduces the dimensionality from 5 to 2 orthogonal principal axes, with essential information conserved during the process. The linear combination of the basis vectors composes a continuous 2D space, where exhaustive mapping rapidly characterizes all possible designs. Figure 7D depicts the mapping results with coupling efficiency larger than 0.7 and back reflection below 15 dB. The authors then select the outranking designs and evaluate them with FDTD full-spectra simulation as a second-stage optimization. All designs perform with 1 dB bandwidth covering the telecommunication C-band ranging from 1530 nm to 1565 nm. The authors demonstrated that the search complexity is reduced by orders of magnitudes, resulting in 400 times less computation time during theoptimization.

**Figure 8:**
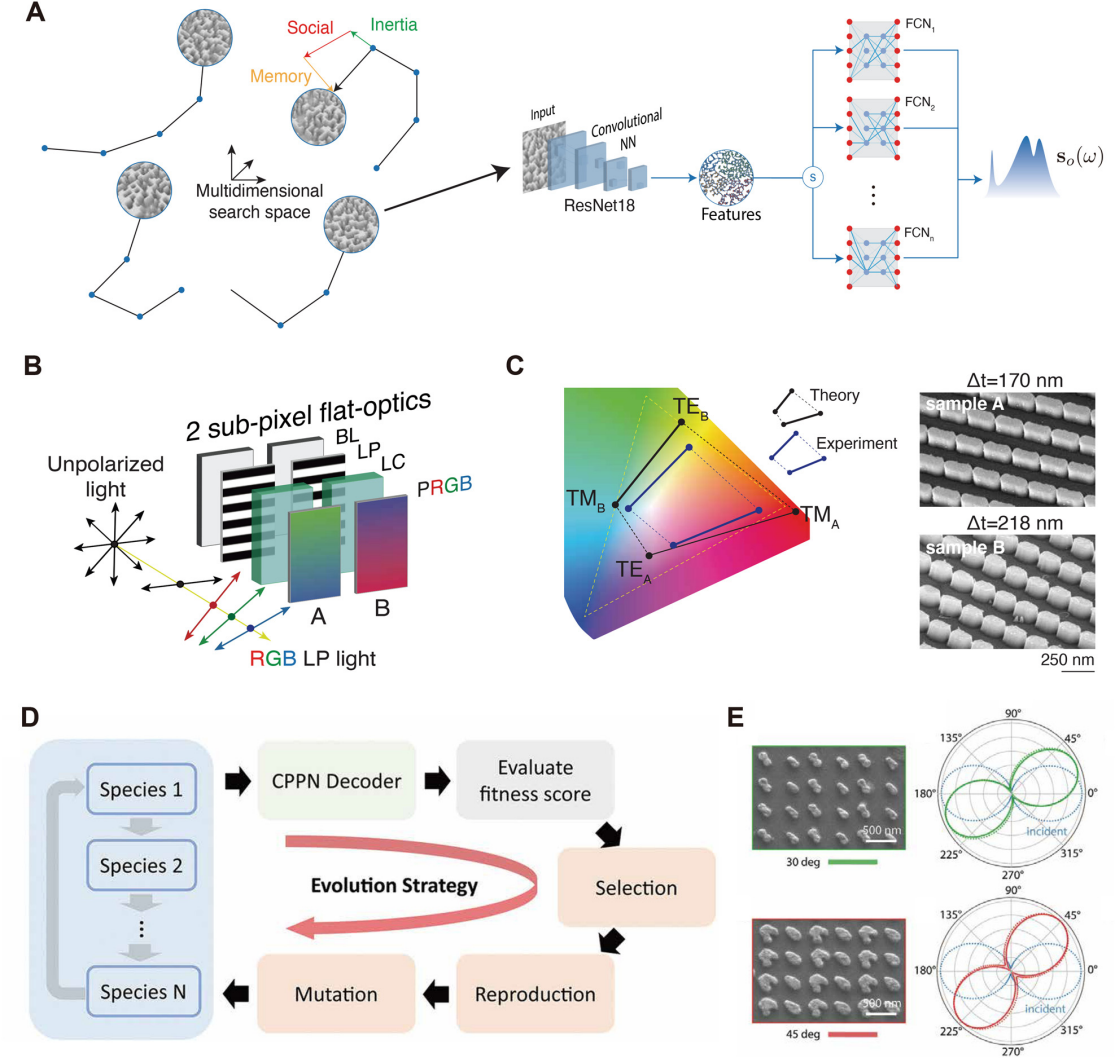
Hybrid design methods leveraging deep learning models as fast EM solver (A) An autonomous learning framework for rule-based evolutionary design, comprising a fast CNN predictor and a parallel PSO algorithm. (B) Generalized two-subpixel color display designed with a polarization-sensitive response, where backlight (BL), linear polarizer (LP), and liquid crystal (LC) layers alter the incident polarization to the flat-optics subpixels. (C) Left: chromaticity gamut of the subpixels in the color space. Right: SEM images of the fabricated subpixels. (A), (B) and (C) are adapted from [18]. (D) A hybrid system for the design of diatomic polarizers including cooperative coevolution algorithm and a pre-trained compositional pattern-producing network. (E) Left: SEM image of two optimized structure with rotation angles of 30° and 45°. Right: incident polarization (blue dash line), desired rotation angle (green/red solid line) and experimental results (green/red dash line). (D) and (E) are adapted from [17].

Nanophotonic inverse design can also help address partial differential equation (PDE)-constrained optimization problems that rely on physical principles. As discussed in the previous section, in recent discriminative inverse design approaches, this idea applies to train networks solving the PDE [127] and approximating the optical response [87, 92]. Another approach utilizes traditional optimization methods as the core unit that produces varying design samples. Getman et al. [18] implemented a hybrid inverse design framework integrating PSO and pre-trained predictors. Focusing on the design and applications surrounding flat-optics surfaces, the authors demonstrated the universal expressivity of these devices from physical principles. The work demonstrates that in suitably engineered nanoresonators, linear propagation and resonances blend, defining a system response *H*(*w*) that can approximate any user-defined function [128]. The design problem of the entire device then reduces to characterizing a combination of nanoresonator shapes. The authors designed and implemented an Autonomous Learning Framework for Rule-based Evolutionary Design (ALFRED), a parallel software exploring a large design space consisting of multimodal nanoresonators described by 2D binary images and discrete thickness values. As depicted in Figure 8A, a parallel swarm optimizer performs the collective search among inertia, social, and memorial elements of designs. Complementing this, an NN reduces the high computational cost of running first-principle EM simulations to a forward propagation within milliseconds during the iterations. A CNN-based, switch-connected simulator network predicts the individual optical responses of devices with various shapes and thicknesses. Two popular example devices, a polarization beam splitter, and a dichroic mirror are fabricated and measured experimentally, showing over 95% transmission efficiency across the visible range.

By using ALFRED, the authors reported a new type of two-subpixel metasurface color display. A conventional LCD color display employs a linear polarizer (LP) sandwich structure, wrapping liquid crystal (LC), and three color filters. The proposed design uses only one LP and LC layer, enabling the free rotation of backlight polarization. Figure 8B shows the schematic, where the unpolarized backlight sources are first polarized by the LP and then rotated by the following LC cell. Two polarization-sensitive metasurfaces filter the output of the LC cell, and through the use of two different chromaticities, compose a displayed color. With this approach, a suitably designed pair of metasurfaces reconstruct a high-fidelity color space similar to RGB displays by manipulating four input variables (i.e., polarization and intensities for two pixels). As shown in Figure 8C, the simulation results and the measured response cover a large gamut area in the chromatic space, indicating a wide color range for displays. In follow-up works [19], the design space expands to combinations of arbitrary polygons, ellipses, and cuboids. Additionally, the training introduces random perturbations on the structure shape to build prediction robustness against fabrication errors. The authors also implemented a t-SNE dimensionality reduction technique and K-means clustering to analyze the predicted responses dynamically. The training applies the data augmentation technique to build robust predictions by absorbing new samples from the high-prediction-error clusters.

The work of Liu et al. [17] also explained the advantage of using NN as a fast EM solver. To design and fabricate diatomic polarizers, the authors developed a hybrid system including a compositional pattern-producing network (CPPN) and a pre-trained simulator network. The CPPN uses convolutional layers, which translate a set of input variables into the pixel value of a device pattern. The generated pattern includes two adequately isolated nanoparticles, namely, meta-atoms as the unit cell of the polarizer. The authors train the simulator network to predict the far-field response as the superposition of responses from two independent meta-atoms. As depicted in Figure 8D, this work applies cooperative coevolution (CC) [129] algorithm to optimize the device structure. Due to the design species represented by the dimensionality-reduced parameterized vectors, the evolution processes fewer variables, thus saving computational efforts. A group of designed meta-atoms output different polarization angles between 15° and 60° with linearly polarized incident lights. Each specific design converges within 20 s on a single-GPU workstation. As a final verification, FEM provides a full-spectra simulation of the optimized design. Figure 8E exemplifies two devices with orientation angles of 30° and 45°. The blue dashed curves indicate the incident polarizations, while the solid line and dashed line in red and green represent the desired rotation angle and measured value, respectively. The fabricated devices show a good approximation to the design object, with angular errors below ±1.3%.

## 5 Conclusions and outlook

In this review, we discussed the start-of-art advances in the field of nanophotonic inverse design. These techniques overcome the limitation of intuition-based designs, boosting the implementation of non-intuitive optical devices, ranging from beam splitters to metalenses, optical cloaks, reflective metasurfaces, and polarization-sensitive displays. These applications have broadened the horizon of how light interacts with complex media, enabling significant leaps towards the miniaturization of optical components and systems for manipulating light.

The first discussed optimization methods represent the oldest and perhaps the most intuitive applications of inverse design. Binary representations and re-parameterization of design structures in topology optimization and nature-based solutions in heuristic methods iterate successive FOM minimization until reaching the optimal solution. Both techniques present different advantages. Topology optimization utilizes straightforward update rules based on fast gradient computation to rapidly converge in local optima of material structures. Heuristics methods, on the contrary, can navigate complex energy landscapes and search for global optima of design structures. The recent exploding interest in artificial intelligence fuels research attention towards inverse designs schemes that leverage data-driven science and engineering. Rather than considering only a particular FOM, different flavors of deep learning methods model complex physical relationships between material properties and measured responses, allowing higher-level FOMs to be optimized, simultaneously. Discriminative learning models serve as universal predictors in the design space, where integrated structures hold the ability to approximate arbitrary optical responses. After being trained on sufficiently large datasets, these methods allow efficient forward simulation and fast design parameters convergence in inverse design schemes. The non-uniqueness problem that commonly exists in such single-model approaches can be solved by subdividing the parameter space and training a series of NN [130]. Generative models further expand the previous architectures’ generalization ability by exploiting a latent space whose projection contains all dominant design factors discovered by the network. Properly trained generative models can produce distributions of optimal designs, sampling various structures and analyzing them concurrently.

A novel trend that is attracting considerable attention is the fusion of optimization schemes with deep learning methods. The precise information flow in topology optimization improves the training stability of NN [125], while the deep learning predictors accelerate the convergence speed of optimization methods significantly [19]. The use of dimensionality reduction methods, including PCA [16], autoencoder-like models [119] and Gaussian mixture models (GMM) [131] facilitates the data-augmentation strategy and overcomes the problems in creating huge datasets. Hybrid methods, in general, incorporate superior features from both optimization and deep learning fields, enabling accurate, robust, and fast-converging design within a computationally efficient framework. We here provide a qualitative comparison in Table 1 of all the mentioned design methods. The table discusses five aspects involving (1) whether the model is differentiable; (2) whether the model is transferable; (3) whether the inverse design is conducted on a distribution of candidates or a single device; (4) the cost of constructing (training/initializing) the model; (5) the cost of producing demanded design (forward prediction/numerical optimization).

**Table 1:** Comparison of inverse design approaches.

	Differentiable	Transferable	Group optimization	Model constructing cost	Production cost
Topology optimization	Yes	No	No	Low	High
Heuristics	No	No	Yes	Medium	High
Discriminative model	Yes	Yes	No	High	Low
Generative model	Yes	Yes	Yes	High	Low
Hybrid model	Yes	Yes	Yes	High	Medium

Nanophotonics inverse design represents a young and exciting research field that proceeds fast, together with the rapid development of resources in artificial intelligence. Future directions in inverse design can occur in implementing new types of large-scale nanophotonics systems in areas where intuition-based design fails to provide efficient solutions. Inverse design techniques can also open the door to implementing structures that direct design typically avoids due to their inherent complexity, either geometrical or material-based. Artificial intelligence can significantly widen this horizon, thanks to the ability to grasp complex input–output relationships from simple data sequences.

A legitimate question is whether the strong permeation of nanophotonics with AI will lessen our understanding of fundamental physical phenomena, as we will rely more and more on approximate models built automatically via machine learning. The evidence gathered in this review supports a positive outcome. In nearly all cases reviewed, the introduction of AI resources accompanies the formulations of novel theories for describing light’s properties or the realization of new hierarchically complex systems that previous work did not explore. As it often happens in science, answering this question will provide sufficient research material for scientists in the present and coming generations.
